# P2X7 receptor signaling promotes inflammation in renal parenchymal cells suffering from ischemia-reperfusion injury

**DOI:** 10.1038/s41419-020-03384-y

**Published:** 2021-01-27

**Authors:** Yingying Qian, Cheng Qian, Kewei Xie, Qicheng Fan, Yucheng Yan, Renhua Lu, Lin Wang, Minfang Zhang, Qin Wang, Shan Mou, Huili Dai, Zhaohui Ni, Huihua Pang, Leyi Gu

**Affiliations:** 1 Department of Nephrology, Molecular Cell Lab for Kidney Disease, Renji Hospital, School of Medicine, Shanghai Jiao Tong University, Shanghai, China; 2 Department of Nephrology, Affiliated Hangzhou First People’s Hospital, Zhejiang University School of Medicine, Hangzhou, China

**Keywords:** Membrane proteins, Mechanisms of disease, Acute kidney injury

## Abstract

Extracellular adenosine triphosphate (ATP) and its receptor, P2X7 receptor (P2X7R), are playing an important role in the pathological process of renal ischemia-reperfusion injury, but their underlying mechanism remains unclear. Also, the effects of tubular epithelium-expressed P2X7 receptor on ischemia acute kidney injury is still unknown. The aim of this study is to clarify if this mechanism involves the activation of nucleotide-binding oligomerization domain-like receptor protein 3 (NLRP3) inflammasome in the renal tubular epithelial cells. In our research, we used male C57BL/6 wild type and P2X7R (−/−) mice, cultured human proximal tubular epithelial cells, and kidneys from acute kidney injury patients. Mice underwent for unilateral nephrectomy combined with the lateral renal pedicle clamping. Cultured cells were subjected to hypoxia/reoxygenation or ATP. Apyrase and A438079 were used to block the extracellular ATP/P2X7 receptor pathway. We also constructed radiation-induced bone marrow (BM) chimeras by using P2X7R (−/−) mice and P2X7R (+/+) wild-type mice. P2X7 receptor deficiency protected from renal ischemia-reperfusion injury and attenuated the formation of NLRP3 inflammasome. By using BM chimeras, we found a partial reduction of serum creatinine and less histological impairment in group wild-type BM to P2X7R (−/−) recipient, compared with group wild-type BM to wild-type recipient. In renal tubular epithelial cells, hypoxia/reoxygenation induced ATP release and extracellular ATP depletion reduced the expression of active IL-1β. ATP activated the NLRP3 inflammasome in renal tubular epithelial cells, which were blunted by transient silence of P2X7 receptor, as well as by P2X7 receptor blocking with A438079. In human samples, we found that patients with Stage 3 AKI had higher levels of P2X7 receptor expression than patients with Stage 1 or Stage 2. Extracellular ATP/P2X7 receptor axis blocking may protect renal tubular epithelial cells from ischemia-reperfusion injury through the regulation of NLRP3 inflammasome.

## Introduction

Acute kidney injury (AKI) is a worldwide public health concern with high rates of incidence and mortality^1^. Ischemia-reperfusion injury (IRI) is a major cause of AKI, leading to tissue injury and robust inflammatory response. It has been well established that molecules from damaged or dying cells, called damage/danger-associated molecular patterns (DAMPs), are triggering the inflammatory response^2,3^. Extracellular adenosine triphosphate ATP (eATP) has been identified as a DAMP, which is released during inflammatory conditions, mechanical deformation, ischemia, and hypoxia. Extracellular ATP exerts its effects by binding to and activating purinergic P2 receptors^4^.

The P2X7 receptor (P2X7R) has an undisputed central role in inflammatory and infectious disorders^5^. The strongest evidence is given by the attribution to the mature and release of IL-1β through the activation of nucleotide-binding domain-like receptor protein 3 (NLRP3) inflammasome in immune cells^6,7^. Koo and colleagues have reported that oxidized ATP, a P2X7R antagonist, protects mice from renal IRI by increasing the number of regulatory T cells (Tregs)^8^. Koo et al. research data indicate an important pro-inflammatory role of P2X7 receptor on ischemia-reperfusion (IR)-induced AKI. However, Tregs infiltrating into the ischemic kidneys were increased after 3 days and 10 days^9^. Early innate immune response has been widely reported to play a fundamental role in renal ischemia-reperfusion injury. In view of this, new studies focusing on initial events triggered by eATP/P2X7R system are required.

In this research, we explore the role of eATP/P2X7 receptor axis on IR-induced AKI, and the contribution of NLRP3 inflammasome to this pathological process. Additionally, we evaluate the renal epithelium-expressed P2X7 receptor actions on kidney’s inflammation and injury during renal IRI.

## Materials and methods

### Animals

All mice protocols were developed in accordance to the standards of the National Institutes of Health Guide for care and use of experimental animals and were approved by the Animal Experimentation Ethics Committee of Renji Hospital, Shanghai Jiaotong University School of Medicine. P2X7R (−/−) mice on a C57BL/6 background were purchased from The Jackson Laboratory (Bar Harbor, ME; stock number: 005576). C57BL/6 mice were obtained from Shanghai SLAC (Shanghai, China).

### Renal ischemia-reperfusion protocol

Six- to eight-week-old male mice were anesthetized with pentobarbital (50 mg/kg) through intraperitoneal injection (i.p.) and the rectal temperature was maintained at 37 °C during surgery. AKI model was established by unilateral nephrectomy combined with the lateral renal pedicle clamping for 35 min. The Sham mice underwent the same surgical procedure except for renal nephrectomy and pedicle clamps that were not conducted. In treatment models, 10 U/25 g Apyrase (Sigma-Aldrich, St. Louis, MO), 200 μmol/Kg A438079 (Tocris Bioscience, Bristol, UK), or phosphate-buffered saline (PBS) were applied intravenously, 30 min after reperfusion. Each group contained five mice. The mice were sacrificed at 24 h after surgery, and the blood, urine, and kidneys were collected for further analysis.

### Bone marrow chimeras

Donor mice were euthanized and bone marrow (BM) cells were collected by flushing tibia and femurs from donors with RPMI 1640 containing 5% fetal bovine serum (Thermo Scientific, Rockford, IL). Six-week-old recipient mice were irradiated with two doses of 6.5 Gy using an X-Ray biological irradiator (Precision X-Ray, North Branford, CT) and were intravenously injected with 5 × 10^6^ donor BM cells at 6 h post irradiation. The chimeric mice (*n* = 4 for each group) were allowed to recover in a sterile environment for 8–10 weeks before the induction of renal ischemia-reperfusion injury.

The chimerism was confirmed by genotyping of genomic DNA from peripheral blood and tails using, respectively, TIANamp Blood DNA Kit (TIANGEN, Beijing, China) and TIANamp Genomic DNA Kit (TIANGEN). The primers for standard polymerase chain reaction (PCR) are shown in Supplementary Table 1.

### Survival experiments

Mice with or without P2X7R deficiency (*n* = 10 for each group) were subjected to unilateral nephrectomy combined with the lateral renal pedicle clamping for 40 min. A 7-day survival was assessed. All mice were sacrificed on day 7.

### Cell culture for HK2 cells

The human proximal tubular epithelial cells (HK2) were cultured in Dulbecco’s modified Eagle’s medium/F12 media (Thermo Scientific) supplemented with 10% fetal bovine serum in a humidified atmosphere of 5% CO_2_.

### Cells stimulation

For hypoxia/reoxygenation (H/R) studies, confluent HK2 cells were incubated under a hypoxic condition of 1% O_2_, 94% N_2_, and 5% CO_2_ in a controlled hypoxic chamber (Thermo Scientific) for 24 h. Then, they were removed to a normoxic condition of 21% O_2_, 74% N_2_, and 5% CO_2_ for reoxygenation. Controls were cultured under a normoxic incubator for identical times. HK2 cells were treated with ATP (Sigma-Aldrich) at indicated concentrations for 8 h. Apyrase (0.2 U/ml, 1 U/ml, or 5 U/ml) or A438079 (20 µM) was added to the culture medium for 1 h before exposing to ATP or H/R. Experiments were performed in triplicate.

### Transient transfection of siRNA

The small interfering RNAs (siRNAs) for P2X7 receptor, NLRP3, and non-specific control were purchased from Thermo Scientific. Cells were cultivated to 50–60% confluence and were then transfected with 30 nM siRNA using RNAiMAX (Thermo Scientific) for 24 h according to the manufacturer’s instructions. The siRNA sequences are as follows: siP2X7R, 5’-GGAGGAAAAUUUGACAUUAtt-3’ and 3’-UAAUGUCAAAUUUUCCUCCgg-5’; siNLRP3, 5’-GGAGAGACCUUUAUGAGAAtt-3’ and 3’-UUCUCAUAAAGGUCUCUCCtg-5’.

### ATP measurement

Mouse urine was collected at 24 h of surgery, separated by centrifugation (1000 × *g*, 10 min, 4 °C), filtered by Millex^®^ Filters with a 0.22 µM pore size (Millipore). Cells were cultured in flat-bottomed, 96-well plates. Cell supernatants were collected, centrifuged (1000 × *g*, 10 min, 4 °C), and filtered. ATP levels were measured immediately with an ENLITEN^®^ ATP Assay System Bioluminescence Detection Kit according to the manufacturer’s instructions (Promega, Madison, WI). The results of urine ATP levels were corrected for urine creatinine excretion.

### Real-time PCR analysis

Total mRNA from kidney tissues and HK2 cells were extracted by using Trizol Reagent (Thermo Scientific) and was reversed transcribed using cDNA reverse transcription kit (TAKARA, Dalian, China). P2X7R, MCP-1, IL-6, NLRP3, ASC transcripts were measured using SYBR Green I reagents (TAKARA) on a thermocycler (LightCycler 480, Roche Applied Science, Mannheim). Comparative C_T_ method^10^ was used and all specific amplicons were normalized to the GAPDH determined under the same conditions. The results were expressed as the target gene/GAPDH mRNA. The sequences of primers (TAKARA) are illustrated in Supplementary Table 2.

### Western blotting

Total protein from kidneys and cells were extracted as previously described^11^. Incubation was carried out with the following primary antibodies: anti-P2X7R (Santa Cruz Biotechnology, Dallas, TX, 1:200), anti-mouse NLRP3 (Adipogen, San Diego, CA, 1:1000), anti-human NLRP3 (Cell Signaling Technology, Beverly, MA, 1:500), anti-ASC (Santa Cruz Biotechnology, Dallas, TX, 1:200), anti-IL-1β (Abcam, Cambridge, MA, 1:500), anti-mouse capsase-1 (Adipogen, 1:1000), anti-human caspase-1 (Cell Signaling Technology, 1:500), anti-human cleaved caspase-1 (Cell Signaling Technology, 1:500, only for Supplementary Fig. 5c), anti-ATP5a (Proteintech, Wuhan, China, 1:1000), anti-ATP5b (Proteintech, 1:1000), anti-Tubulin (Abcam, 1:2000), and anti-GAPDH (Cell Signaling Technology, 1:2000). After incubation with the appropriate horseradish peroxidase-conjugated IgG (Cell Signaling Technology), specific signals were determined on a Tanon 5200 Chemiluminescent Imaging System (Tanon, Shanghai) by using an ECL kit (Thermo Scientific).

### Immunochemistry and immunofluorescent staining

Kidney tissues were fixed in 4% paraformaldehyde, paraffin embedded, and incubated with the primary antibodies against caspase-1 (p20) (Adipogen, 1:100), ATP5a (Proteintech, 1:100), ATP5b (Proteintech, 1:100), and P2X7R (Thermo Scientific, 1:100). Then, sections were incubated in horseradish peroxidase-conjugated secondary antibody. Hematoxylin was used as a counterstain. After DAB reaction, slices were examined under a microscope (Leica).

For immunofluorescent staining, kidneys were fixed in 4% paraformaldehyde, dehydrated in 20% sucrose, embedded with OCT (Tissue Tek, Sakura, Japan), and cut by a cryostat. For staining of Ly6G, rat anti-mouse Ly6G (Abcam, 1:100) antibody was used. For double staining of NLRP3 with P2X7R, NLRP3 with ASC, and NLRP3 with IL-1β, primary antibodies with goat anti-mouse NLRP3 (Abcam, 1:100), rabbit anti-mouse P2X7R (Thermo Scientific, 1:100), rabbit anti-mouse ASC (CST, 1:100), rabbit anti-mouse IL-1β (Abcam, 1:100) were used. For double staining of P2X7R with IL-1β, P2X7R with E-cadherin, and P2X7R with CD11b, primary antibodies of rabbit anti-mouse P2X7R (Thermo Scientific, 1:100) and goat anti-mouse IL-1β (R&D systems, Minneapolis, 1:400), rat anti-mouse E-cadherin (Abcam, 1:200), rat anti-mouse CD11b (Abcam, 1:200) were used. For double staining of NLRP3 with E-cadherin, and NLRP3 with CD11b, mouse anti-mouse NLRP3 (Adipogen, 1:100), rat anti-mouse E-cadherin (Abcam, 1:200), rat anti-mouse CD11b (Abcam, 1:200) were used. Then, frozen sections were incubated in the fluorescent-conjugated secondary antibodies (Abcam) and analyzed on a microscope (Leica).

### Patients

AKI patients were identified using KDIGO definition and they underwent renal biopsy at the Department of Nephrology in East District of Renji Hospital, between January 1st, 2013 to December 31th, 2017. The kidney sections were examined by two renal pathologists blinded to the clinical data. A total of 33 patients with renal pathological manifestations of acute tubular lesions were enrolled in the study. Among them, patients (*n* = 10) with a baseline Scr of more than 110 μmol/L were excluded. The positive areas for P2X7R were calculated by a researcher who was blinded to the study using Image-Pro Plus software (Media Cybernetics, Rockville, MD). This study was approved by the ethical review board of Renji Hospital, School of Medicine, Shanghai Jiaotong University. All of the patients were given and accepted informed consent form prior to their enrollment.

### Statistical analysis

Mice with poor physical condition were excluded before grouping. We used random number method for random allocation. Quantitative data are representative of at least three experiments. Data are presented as mean ± s.e.m. and were subjected to unpaired Student’s t test for normal distribution. Non-normally distributed continuous data are presented as medians with interquartile range and compared using Mann-Whitney U test. The survival data were analyzed by the Kaplan-Meier test. Analyses were made using GraphPad Prism 7.0 software. A *p* value of < 0.05 was considered significant.

## Results

### Renal ischemia-reperfusion injury induces the release of ATP and upregulates the expression of P2X7 receptor in HK2 cells

**Fig. 1:**
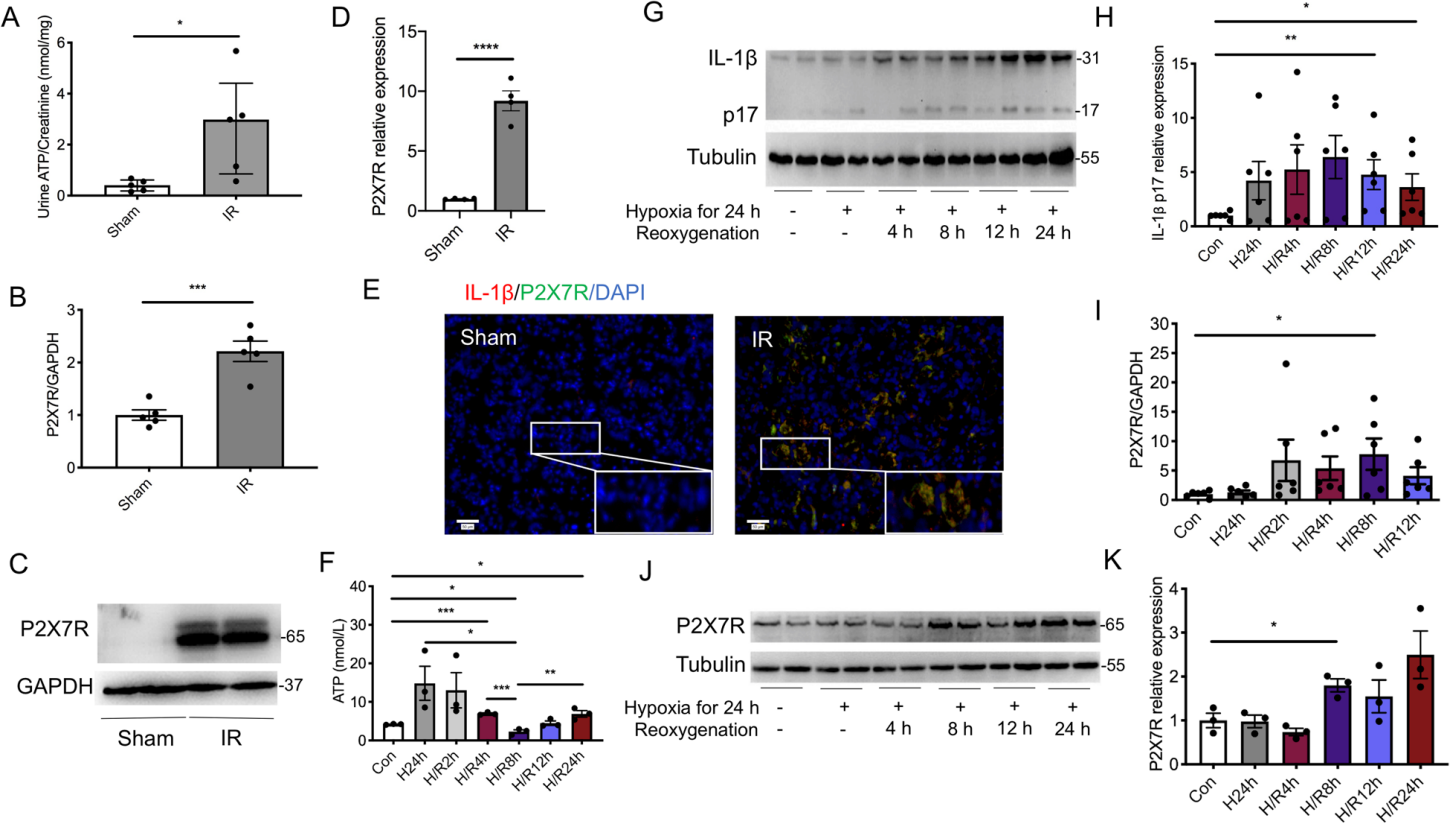
Renal ischemia-reperfusion injury induced the release of ATP and upregulated the expression of P2X7 receptor in HK2 cells. A total of ten wild-type mice were randomly divided into the group Ischemia-Reperfusion (IR) (*n* = 5) and the group Sham (*n* = 5). **A** The levels of urine ATP, corrected for urine creatinine excretion. **B** The levels of P2X7 receptor mRNA in kidneys were measured by quantitative polymerase chain reaction. **C** Representative immunoblots and **D** aggregate densitometric quantification of whole kidney lysate for P2X7 receptor expression. **E** Representative immunofluorescent staining for IL-1β (red) and P2X7R (green) merged with 4’, 6-diamidino-2-phenylindole (DAPI, blue). HK2 cells were subjected to hypoxia for 24 h followed by reoxygenation for a determined time. **F** The time course for ATP concentrations in the supernatants. **G** Representative immunoblots and **H** aggregate densitometric quantification of cell lysate for IL-1β p17 expression. **I** The levels of P2X7 receptor mRNA in HK2 cells. **J** Representative immunoblots and **K** aggregate densitometric quantification of HK2 cell lysate for P2X7 receptor expression. *p < 0.05, **p < 0.01, ***p < 0.001, ****p < 0.0001.

Renal IRI dramatically induced renal dysfunction and histological damage at 24 h post-reperfusion (Supplementary Fig. 1A, B). To evaluate the release of ATP after renal IRI, the concentrations of urine ATP were detected. AKI mice had significantly higher levels of urine ATP than sham-operated mice [2.98 (0.85, 4.41) nmol/mg vs. 0.41 (0.18, 0.61) nmol/mg, *p* < 0.05] (Fig. 1A). However, no difference was found in the expression of ATP synthases between AKI and Sham groups (Supplementary Fig. 1C–E). We also found increased gene transcription and protein production of ATP receptor, P2X7 receptor, in AKI mice (Fig. 1B–D). P2X7 receptor protein was expressed primarily in tubular epithelial cells, and was co-localized with IL-1β after renal IRI (Fig. 1E).

In vitro, with human proximal renal tubular epithelial cells (HK2), the levels of extracellular ATP were increased at 24 h of hypoxia, then started to recover following reoxygenation (Fig. 1F). The levels of active IL-1β proteins were also increased after hypoxia/reoxygenation (H/R) stimulating, the peaks of which were behind the release of ATP (Fig. 1G, H). The transcription of P2X7 receptor mRNA was upregulated in H/R groups and reached the highest level at 8 h after reoxygenation, which was 7.8 times higher than that in the control group (Fig. 1I). Immunoblotting showed similar findings, with the expression of P2X7 receptor protein increasing significantly at 8 h after H/R, by 1.8 times compared with the controls (Fig. 1J, K).

### Extracellular ATP/P2X7 receptor contributes to the upregulation of active IL-1β expression in response to renal ischemia-reperfusion injury

**Fig. 2:**
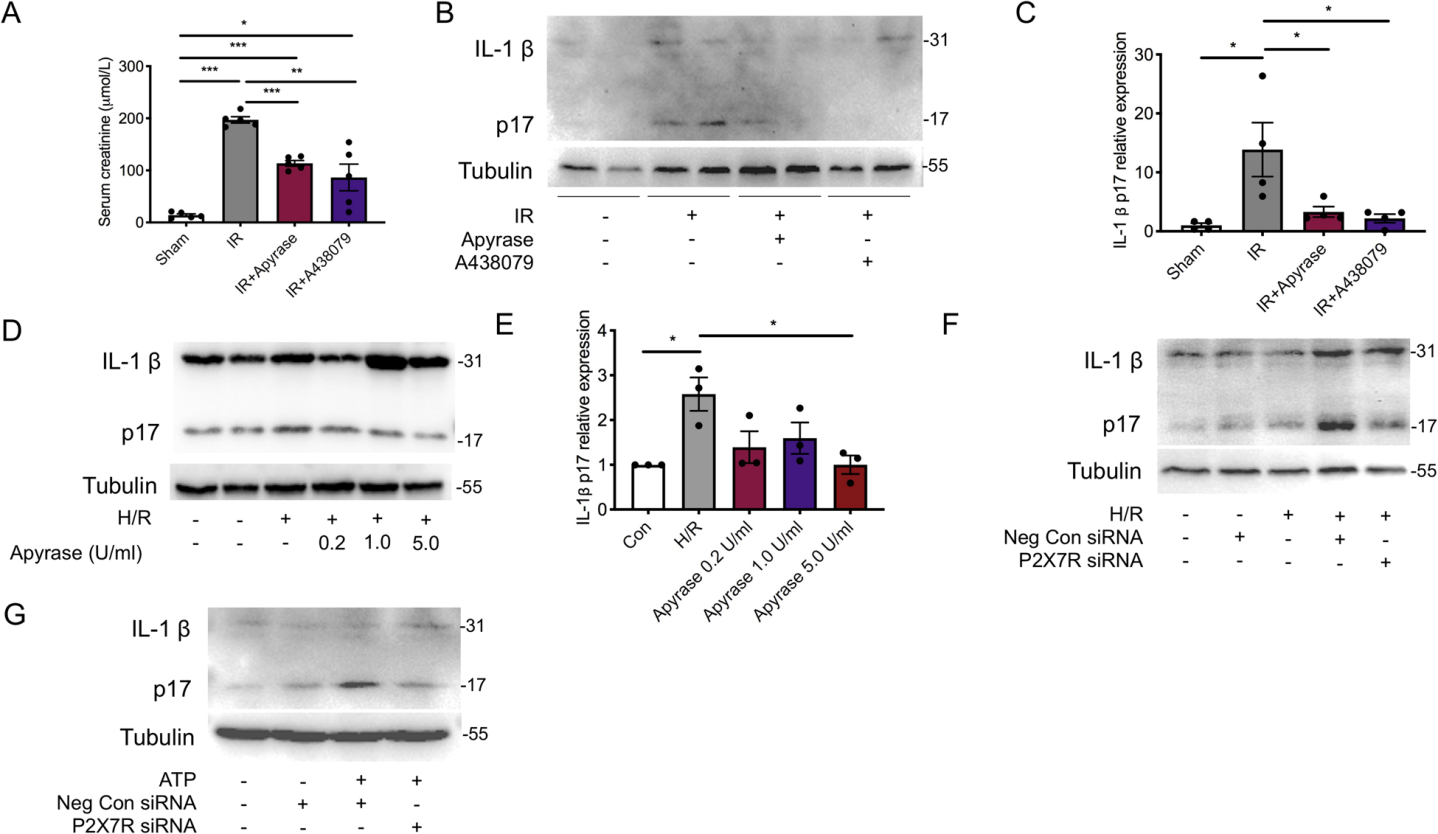
Extracellular ATP/P2X7 receptor contributed to the upregulation of active IL-1β expression in response to renal ischemia-reperfusion injury. A total of twenty wild-type mice were randomly divided into the Sham, Ischemia-Reperfusion, Ischemia-Reperfusion + Apyrase, and Ischemia-Reperfusion + A438079 groups, and were intravenously applied with phosphate-buffered saline, Apyrase, or A438079 at 30 min post-reperfusion accordingly. **A** The concentrations of serum creatinine were measured. **B** Representative immunoblots and **C** aggregate densitometric quantification of whole-kidney lysate for IL-1β p17 expression. HK2 cells were subjected to **D**–**F** hypoxia for 24 h followed by reoxygenation for 8 h or to **G** ATP at 4 mM, with or without indicated pretreatment. **D**, **F**, **G** Representative immunoblots and **E** aggregate densitometric quantification of cell lysate for IL-1β p17 expression. *p < 0.05, **p < 0.01, ***p < 0.001.

We next investigated the role of ATP/P2X7 receptor signaling in the activation of IL-1β in renal tubular epithelial cells. Firstly, we displayed significantly lower levels of serum creatinine in AKI mice treated with Apyrase (an ATPase) or A438079 (a P2X7R inhibitor) compared to that in non-treatment mice (Fig. 2A). Then, we found that the upregulated expression of renal active IL-1β protein in AKI mice was also prohibited by Apyrase or A438079 treatments (Fig. 2B, C).

In HK2 cells, H/R upregulated the expression of active IL-1β protein by 2.5 times, which was ameliorated by application of Apyrase (Fig. 2D, E). To investigate the role of P2X7 receptor, transient genetic downregulation of P2X7 receptor was used. Transfection of P2X7 receptor siRNA reduced the expression of P2X7 receptor protein by 69% and 84% at, respectively, 48 h and 72 h post-transfection (Supplementary Fig. 2). We showed that the expression of active IL-1β protein in H/R-suffered cells with negative control siRNA (Neg Con siRNA) transfection was higher than that transfected with P2X7 receptor siRNA (Fig. 2F). In line with this, ATP induced the expression of active IL-1β protein in HK2 cells, which was attenuated by P2X7 receptor silence (Fig. 2G).

### P2X7 receptor expressed on renal radioresistant cells partially contributes to renal ischemia-reperfusion injury

**Fig. 3:**
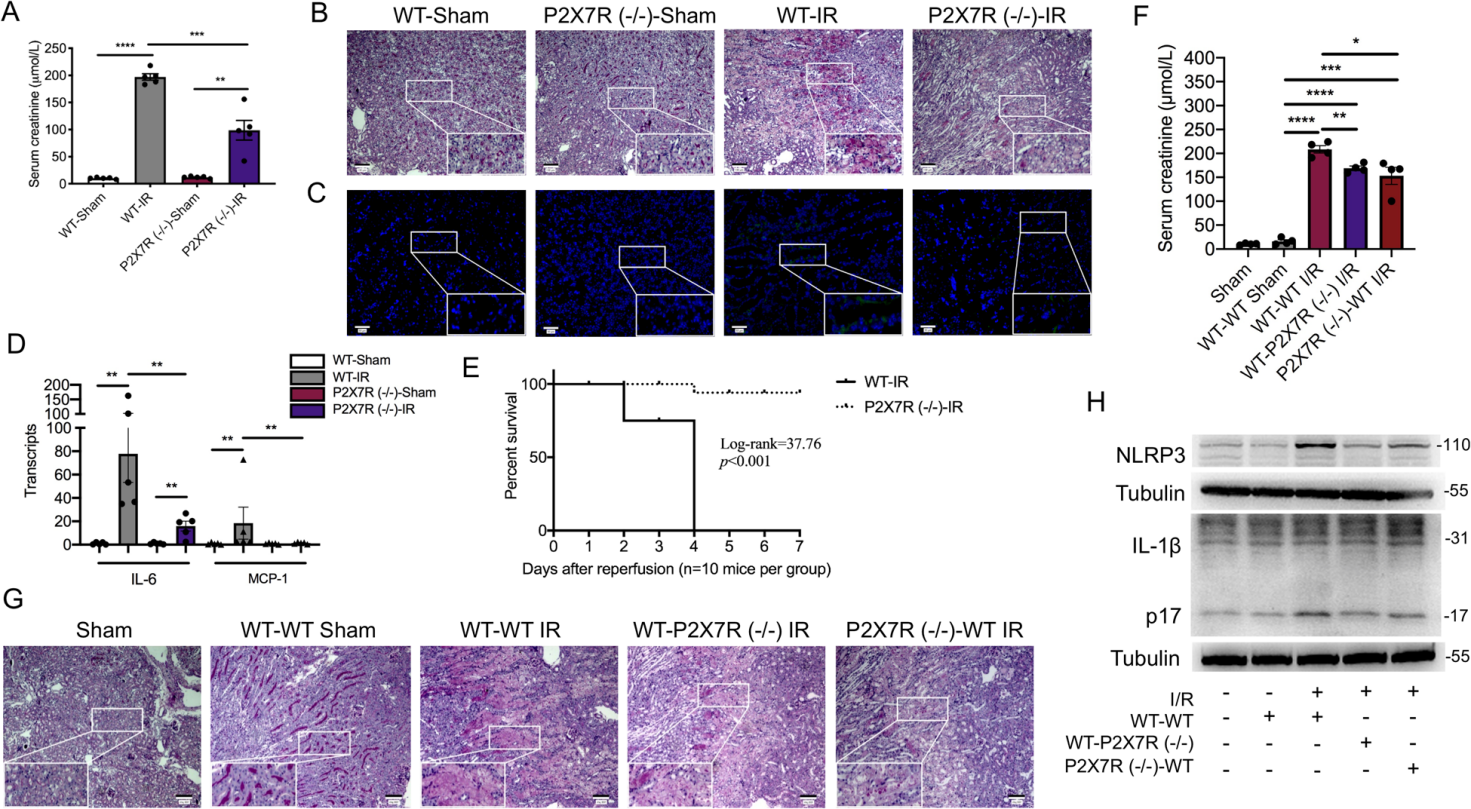
P2X7 receptor gene knockout protected from renal ischemic-reperfusion injury. **A**–**D** Male C57BL/6 wild-type mice and P2X7 receptor (−/−) counterparts were subjected to renal ischemic-reperfusion or sham operation (*n* = 5 per group). **A** The concentrations of serum creatinine. **B** Periodic acid Schiff staining of kidney sections. **C** Representative immunofluorescent staining for Ly6G (green) merged with DAPI. **D** The levels of IL-6 and MCP-1 mRNA by real-time polymerase chain reaction. **E** The survival analysis for renal ischemic-reperfusion injury between wildtype and P2X7 receptor (−/−) mice (*n* = 10 mice each group). **F**–**H** We constructed radiation-induced bone marrow chimeras between P2X7 receptor (−/−) and P2X7 receptor (+/+) mice. The mice were grouped into the wild-type bone marrow to wild-type recipient (WT–WT), the wild-type bone marrow to P2X7 receptor (−/−) recipient (WT–P2X7R (−/−)), and the P2X7 receptor (−/−) bone marrow to wild-type recipient (P2X7R (−/−)-WT) (*n* = 4 per group), and then were subjected to renal ischemic-reperfusion injury or sham operation. **F** The concentrations of serum creatinine were measured. **G** Representative Periodic acid Schiff staining of kidney sections. **H** Representative immunoblots of whole-kidney lysate for the expression of IL-1β p17 and NLRP3 proteins. *p < 0.05, **p < 0.01, ***p < 0.001, ****p < 0.0001.

To further confirm the critical role of P2X7 receptor in renal ischemic-reperfusion injury, P2X7 receptor gene-null (P2X7R (−/−)) mice were used. As showed in Fig. 3A, P2X7R (−/−)-IR mice had significantly lower levels of serum creatinine than that of WT-IR mice (97.20 ± 17.06 μmol/L vs. 197.00 ± 6.01 μmol/L, *p* < 0.001). Deficiency of P2X7 receptor attenuated the histological damage and decreased the infiltration of Ly6G-positive cells in kidneys induced by renal IRI (Fig. 3B, C). The transcripts of pro-inflammatory cytokines such as MCP-1 and IL-6 mRNA expression were significantly lower in P2X7 receptor gene-null mice than that in wild-type mice after suffering from renal IRI (Fig. 3D). We also analyzed the survival rates. Wild-type mice suffering from renal IRI all died on day 4 (*n* = 10), while eight out of ten mice with P2X7 receptor deficiency survived at day 7 (*p* < 0.001, Fig. 3E).

We found that the expression of P2X7R and NLRP3 proteins increased after IRI, and that the proteins were co-localized with both CD11b and E-cadherin (Supplementary Fig. 3). Next, we investigate whether P2X7 receptor on renal radioresistant cells participates in renal IRI. Thus, we performed the BM chimeras between P2X7R (−/−) and P2X7R (+/+) mice. We confirmed the various chimeras by PCR for *P2X7r* gene in their radioresistant tails and their radiosensitive blood cells (Supplementary Fig. 4). We found a partial reduction of serum creatinine and an ameliorated histological damage in AKI mice of both group P2X7R (+/+) BM to P2X7R (−/−) recipient and group P2X7R (−/−) BM to P2X7R (+/+) recipient, compared with group P2X7R (+/+) BM to P2X7R (+/+) recipient (Fig. 3F, G). The expression of active IL-1β and NLRP3 proteins induced by renal IRI was also attenuated in group P2X7R (+/+) BM to P2X7R (−/−) recipient and group P2X7R (−/−) BM to P2X7R (+/+) recipient (Fig. 3H).

### P2X7 receptor contributes to renal ischemia-reperfusion injury by activating NLRP3 inflammasome

**Fig. 4:**
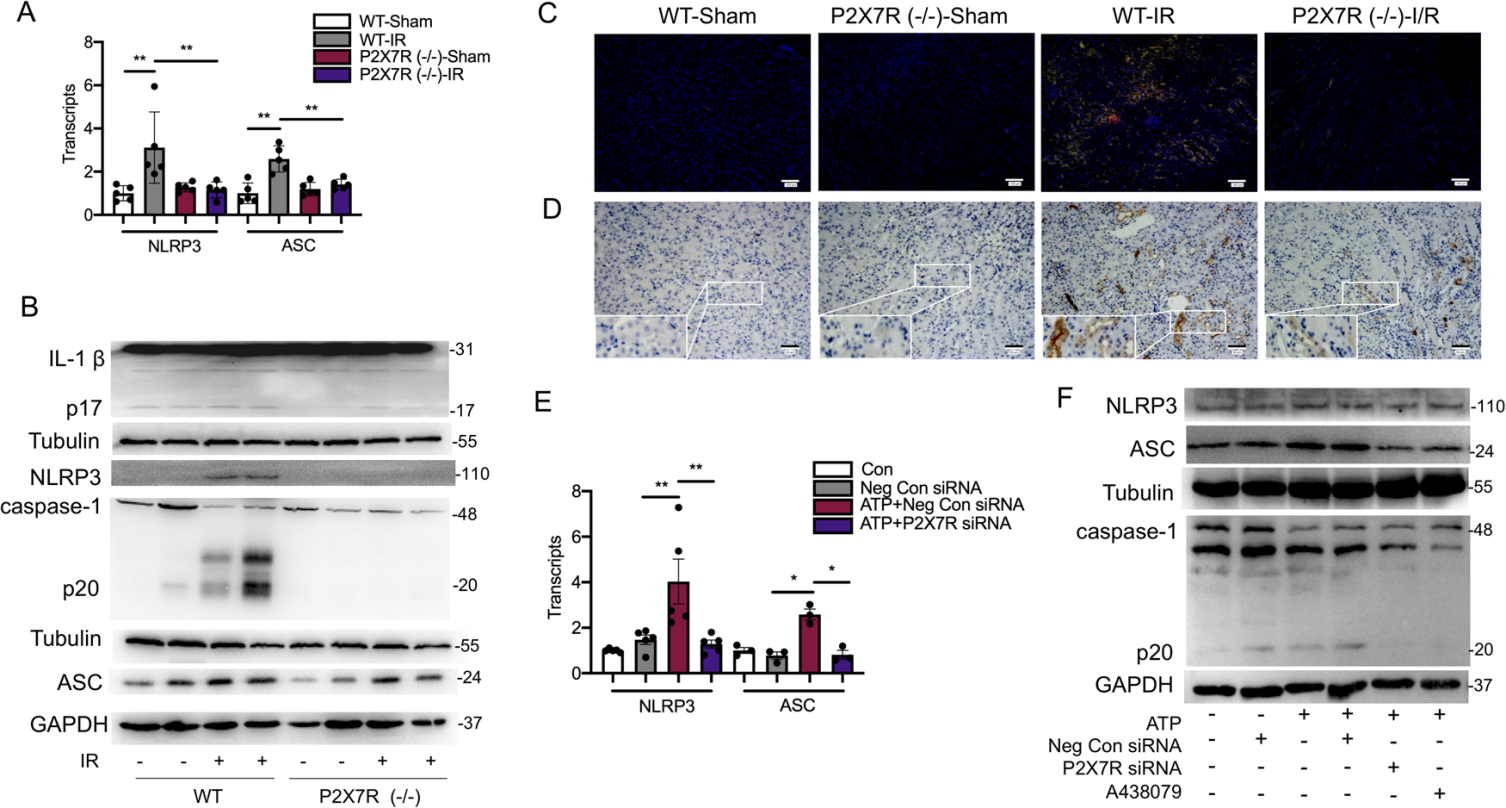
NLRP3 inflammasome was inhibited by P2X7 receptor deficiency during renal ischemic-reperfusion injury. For **A** to **D**, male C57BL/6 wild-type mice and P2X7 receptor (−/−) counterparts were subjected to renal ischemic-reperfusion or sham operation (*n* = 5 per group). **A** The levels of NLRP3 mRNA and ASC mRNA by real-time polymerase chain reaction. **B** Representative immunoblots of whole-kidney lysate for the expression of NLRP3 inflammasome associated proteins (IL-1β p17, NLRP3, cleaved caspase-1, and ASC). **C** Representative immunofluorescent staining for IL-1β (green) and NLRP3 (red) merged with DAPI (blue), and **D** immunochemistry for cleaved caspase-1. For **E** and **F**, HK2 cells were subjected to ATP at 4 mM, and were transfected -or not- with negative control siRNA (Neg Con siRNA) or P2X7 receptor siRNA, or treated with A438079. **E** The levels of NLRP3 mRNA and ASC mRNA were measured by real-time polymerase chain reaction. **F** Representative immunoblots of cell lysate for the expression of NLRP3, ASC, cleaved caspase-1 proteins in response to ATP at 4 mM. A438079 was applied to block P2X7 receptor at 20 µM. *p < 0.05, **p < 0.01.

The activation of IL-1β is controlled by the inflammasome-dependent pathway^12^. Among NLR inflammasome complexes, the NLRP3 inflammasome has been the most widely characterized. We hence explored if NLRP3 inflammasome was responsible for ATP/P2X7 receptor-induced expression of active IL-1β and the followed renal injury. Immunostaining results displayed a co-localization of NLRP3 with P2X7 receptor (Supplementary Fig. 5A), and NLRP3 with ASC (Supplementary Fig. 5B), mainly in the junction of cortex and medulla, after the renal IRI. Ischemic-reperfusion injury increased the levels of NLRP3 mRNA and ASC mRNA in kidneys, which were significantly lower in P2X7R (−/−) mice than those in wild-type mice (Fig. 4A). Immunoblotting data demonstrated that the NLRP3 inflammasome associated proteins (NLRP3, ASC, cleaved caspase-1, cleaved IL-1β) were also increased in wild-type AKI kidneys, but were downregulated in AKI kidneys with P2X7 receptor gene knockout (Fig. 4B). Histological data confirmed lower expressions of NLRP3, IL-1β (Fig. 4C), and cleaved caspase-1 (Fig. 4D) in renal tubular epithelial cells in P2X7 receptor deficiency mice, compared to those in wild-type mice suffering from renal IRI.

In vitro with HK2 cells, we found increased expressions of NLRP3, cleaved caspase-1, and ASC proteins, after suffering from H/R and after ATP stimulation (Supplementary Fig. 5C, D). The levels of NLRP3 and ASC transcripts were upregulated in cells transfected with negative control siRNA, when applied with ATP, but were ameliorated in cells transfected with P2X7R siRNA (Fig. 4E). By using immunoblotting assay, we showed that the upregulation of NLRP3, ASC, and cleaved caspase-1 proteins induced by ATP, was attenuated by P2X7 receptor silencing, as well as by P2X7 receptor antagonist (Fig. 4F).

### The expression of renal P2X7 receptor is increased in patients with stage 3 AKI

**Fig. 5:**
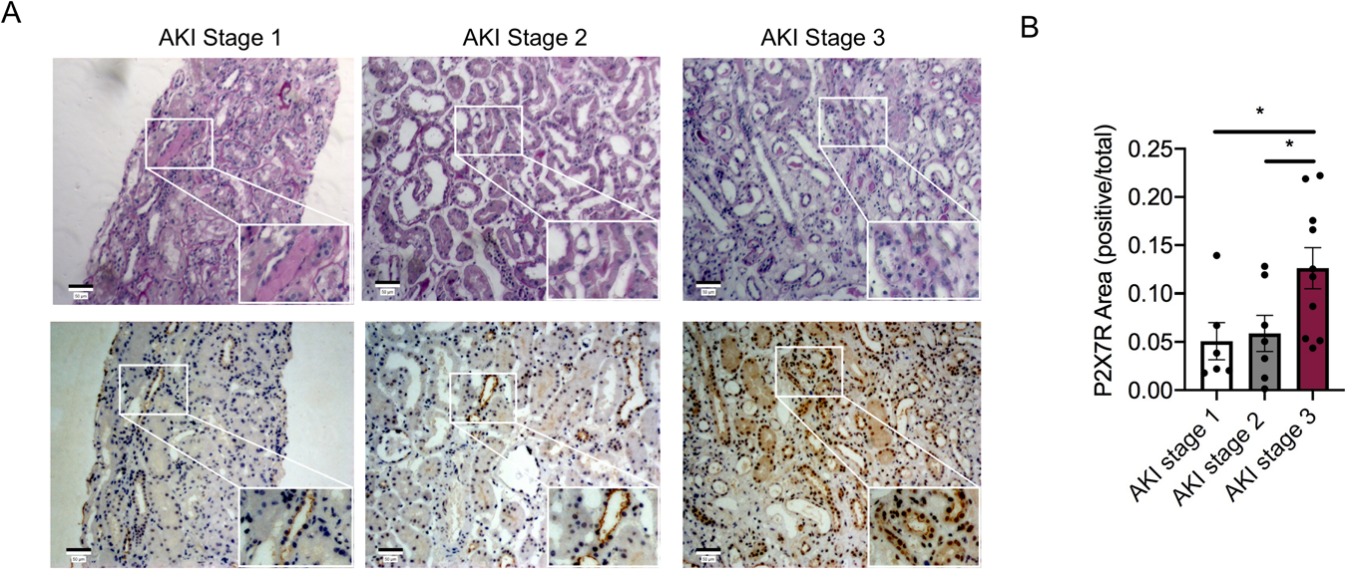
The renal expression of P2X7 receptor was increased in patients with AKI stages 3. **A** PAS staining (top) and representative immunochemistry for P2X7 receptor (bottom) in human kidneys. **B** The positive areas for P2X7 receptor were calculated by a researcher who was blinded to the present study using Image-Pro Plus software. *p < 0.05.

To assess the expression of P2X7 receptor in patients with AKI, we screened the patients who were diagnosed as AKI using KDIGO definition and underwent renal biopsy in the Department of Nephrology in Renji Hospital, School of Medicine, Shanghai Jiaotong University. Among them, 33 patients with renal pathological manifestations of acute tubular lesions were enrolled in the study. Ten patients were excluded, as their baseline serum creatinine was more than 110 μmol/L. As shown in Supplementary Table 3, a total of 23 patients finally participate into the study and were grouped into AKI stage 1, 2, and 3 according to KDIGO guidelines. We found that patients in Stage 3 had higher levels of P2X7 receptor protein expression compared to that of patients in Stage 1 and Stage 2 (Fig. 5A, B).

## Discussion

Inflammation plays a major role in the pathophysiology of IR-induced AKI^13,14^. Since the “Danger Theory” has been proposed by Matzinger^15^, several DAMPs, including ATP, have been identified as ways to trigger the immune system. This concept is widely used to explain the sterile inflammation mechanism during ischemic-reperfusion injury. Here, we observed an increased generation of urine ATP following renal IRI and an increased release of ATP in hypoxic HK2 cells, which functions through binding to and activating P2X7 receptor. We further solidified the emerging axis between eATP/P2X7 receptor and the induction of IL-1β in renal IRI, and we suggested that the formation of NLRP3 inflammasome is important in relation to the pro-inflammatory effects of the eATP/P2X7 receptor axis in ischemic tubular cells. P2X7 receptor, in both tubular epithelial cells and immune cells, played a key role in IR-induced AKI.

The concentration of extracellular ATP is controlled: only a small fraction of ATP is released from the intact cells and it is rapidly hydrolyzed by CD39 and CD73^16^. Our findings demonstrated an increased accumulation of ATP in the extracellular compartment of hypoxic epithelial tubular cells, whereas no change was found in the ATP synthase expression. It is possible that cell release was the main reason underlying the increased levels of extracellular ATP, other than synthase enhancement. Reducing eATP by ATPases protected from renal IRI and downregulated the expression of active IL-1β. Although previous studies have reported a renoprotective effect of extracellular ATP depletion^17^, our data are strengthening the hypothesis that eATP, released by injured epithelial tubular cells, exerted a pro-inflammatory role by activating P2X7 receptor.

P2X7 receptor was originally characterized in immune cells, where P2X7 receptor blocking showed therapeutic effects on several inflammatory diseases, including rheumatoid arthritis, inflammatory bowel disease, and allograft rejection^18,19^. P2X7 receptor has recently been found expressed in wide cell types, especially in epithelial cells^20^. In the present study, we showed that a small amount of P2X7 receptor was expressed in renal tubular epithelial cells at physiologic conditions, but was significantly upregulated following ischemic insult. In vitro with tubular epithelial cells, we found that P2X7 receptor silence reduced the production of cleaved IL-1β induced by ATP or H/R, and inhibited the activation of NLRP3 inflammation. We used bone marrow chimeras between P2X7R (+/+) and P2X7R (−/−) mice to evaluate the contribution of P2X7 receptor on BM-derived cells and on renal parenchymal cells. We suggested that in both tubular epithelium and immune cells, P2X7 receptor is contributing to the pathophysiological process of renal IRI showing a 19% reduction of serum creatine in P2X7R (+/+) BM to P2X7R (−/−) recipient group and 27% reduction of that in P2X7R (−/−) BM to P2X7R (+/+) recipient group, compared with P2X7R (+/+) BM to P2X7R (+/+) recipient group. These results were also supported by the immunofluorescence evidence showing that IRI increased the expression of P2X7R protein, both in immune cells and in renal tubules.

Koo and colleagues were the first to demonstrate an anti-inflammatory role of P2X7 receptor antagonist, oxidized ATP, on IR-induced AKI mouse model by increasing the number of Tregs^8^. However, a significant trafficking of Tregs into the ischemic kidneys occurs after 3 days and 10 days^9^, and the most serious kidney impairment happens at 24 h post-reperfusion^11^. Thus, an early and initial event that senses the danger signals can be of a great importance. Inflammasomes are intracellular complexes comprising an inflammasome sensor molecule, the adapter protein ASC and caspase-1^21^. The formation of inflammasomes is responsible for the maturation and secretion of IL-1β cytokine family, leading in turn to the infiltration and the activation of other immune cells, such as neutrophils, and the adaptive effector T cells^22,23^. NLRP3 inflammasome is shown to be a key complex for eATP/P2X7 receptor induced IL-1β activation in neutrophil^6^, macrophage^24^, and astrocyte^7^. In fact, tubular epithelium is not merely a passive victim of ischemic insult, but is actively contributing to even greater injury^25^. Recent studies have suggested that epithelium purine signaling pathway plays an important role in tissue inflammation, including salivary gland epithelium^20^ and liver cells^26^. To our knowledge, this study is the first to explore whether tubular epithelium eATP/P2X7 receptor signaling plays an active role in renal inflammation by eliciting NLRP3 inflammasome. Besides the co-expression of P2X7 receptor with NLRP3 inflammasome in post-ischemic tubules, we further demonstrated that both extracellular ATP depletion and P2X7 receptor antagonist are improving ischemic AKI and inhibiting NLRP3 inflammasome. P2X7 receptor deficiency rescued AKI by displaying an improvement in renal function, a reduced NLRP3 inflammasome activation, and a less production of inflammatory factors including IL-1β, IL-6, and MCP-1, in P2X7 receptor-null mice compared with wild-type mice. These data are indicating that by lowering the activity or reducing the amounts of P2X7 receptor, or even decreasing the amounts of its ligands, may impact NLRP3 inflammasome activation and mitigate ischemic AKI.

Above all, these data are indicating that purinergic signaling in tubular epithelial cells, through mediating the activation of NLRP3 inflammasome, played a role in renal inflammation and injury induced by ischemic-reperfusion. P2X7R, on both BM-derived and renal parenchymal cells, is required for renal IRI.

## Supplementary information

Supplementary Tables

Supplementary Figure legends

Supplementary figure 1

Supplementary figure 2

Supplementary figure 3

Supplementary figure 4

Supplementary figure 5

## Supplementary information

**Supplementary information** The online version contains supplementary material available at (10.1038/s41419-020-03384-y).
