# Supplementary material for: P2X7 receptor signaling promotes inflammation in renal parenchymal cells suffering from ischemia-reperfusion injury
Source: Cell Death Dis. 2021 Jan 27;12(1):132. doi: 10.1038/s41419-020-03384-y (PMC7841183; doi:10.1038/s41419-020-03384-y)
Supplement: Supplementary file 1 — Supplementary Tables [file 41419_2020_3384_MOESM1_ESM.doc]

**Supplementary Table 1. Primers for Standard polymerase chain reaction.**

| Gene | Primer Sequence |
| --- | --- |
| *P2x7r* | Wild type Forward: 5’-TCACCACCTCCAAGCTCT TC-3’  Common: 5’-TATACTGCCCCTCGGTCTTG-3’ |
|  | Mutant Forward: 5’-GCCAGAGGCCACTTGTGTAG-3’ |

**Supplementary Table 2. Primers for Real-Time polymerase chain reaction.**

| Gene | Primer Sequence |
| --- | --- |
| *H-gapdh* | Forward: 5’- ACAACTTTGGTATCGTGGAAGG -3’  Reverse: 5’- GCCATCACGCCACAGTTTC-3’ |
| *M-gapdh* | Forward: 5’-CCAATGTGTCCGTCGTGGATCT-3’  Reverse: 5’-GTTGAAGTCGCAGGAGACAACC-3’ |
| *H-p2x7r* | Forward: 5’-TATGAGACGAACAAAGTCACTCG-3’  Reverse: 5’-GCAAAGCAAACGTAGGAAAAGAT-3’ |
| *M-p2x7r* | Forward: 5’-GACAAACAAAGTCACCCGGAT-3’  Reverse: 5’-CGCTCACCAAAGCAAAGCTAAT-3’ |
| *H-nlrp3* | Forward: 5’-CCACAAGATCGTGAGAAAACCC-3’ |
|  | Forward: 5’-CGGTCCTATGTGCTCGTCA-3’ |
| *M-nlrp3* | Forward: 5’-ATTACCCGCCCGAGAAAGG-3’ |
|  | Forward: 5’-TCGCAGCAAAGATCCACACAG-3’ |
| *H-asc* | Forward: 5’-TGGATGCTCTGTACGGGAAG-3’ |
|  | Forward: 5’-CCAGGCTGGTGTGAAACTGAA-3’ |
| *M-asc* | Forward: 5’-CTTGTCAGGGGATGAACTCAAAA-3’ |
|  | Forward: 5’-GCCATACGACTCCAGATAGTAGC-3’ |
| *M-il-6* | Forward: 5’-TAGTCCTTCCTACCCCAATTTCC-3’ |
|  | Forward: 5’-TTGGTCCTTAGCCACTCCTTC-3’ |
| *M-mcp-1* | Forward: 5’-CACCTGCTGCTACTCATTC-3’ |
|  | Forward: 5’-CTTCAGATTTACGGGTCAAC-3’ |

H, human; M, mouse.

**Supplementary Table 3 Characteristics of patients with acute kidney injury.**

|  | All  (n=23) | Stage1  (n=6) | Stage2  (n=7) | Stage3  (n=10) | *p* value |
| --- | --- | --- | --- | --- | --- |
| Male, n (%) | 16 (65.22%) | 6 (100%) | 3 (42.86%) | 6 (60%) |  |
| Age (year) | 39.09 ± 17.83 | 45.17 ± 15.90 | 33.00 ± 11.12 | 39.70 ± 22.36 | 0.487 |
| Baseline Scr | 80.47 ± 15.07 | 93.00 ± 12.20 | 76.91 ± 17.38 | 75.44 ± 11.33 | 0.051 |
| Scr at biopsy | 273.08 ± 157.67 | 154.70 ± 27.84 | 198.53 ± 45.91 | 396.30 ± 168.52 | 0.001 |

Scr, serum creatinine.
