# Supplementary material for: P2X7 receptor signaling promotes inflammation in renal parenchymal cells suffering from ischemia-reperfusion injury
Source: Cell Death Dis. 2021 Jan 27;12(1):132. doi: 10.1038/s41419-020-03384-y (PMC7841183; doi:10.1038/s41419-020-03384-y)
Supplement: Supplementary file 2 — Supplementary Figure legends [file 41419_2020_3384_MOESM2_ESM.pdf]

Supplementary Figure 1. No difference was found in ATP synthases expression between AKI and Sham groups. (A) The concentrations of serum creatinine (Scr) and blood urea nitrogen (BUN) were measured between the group Ischemia-Reperfusion (n=5) and the group Sham (n=5). (B) Periodic acid Schiff staining of kidney sections. (C) Representative immunoblots and (D) aggregate densitometric quantification of whole-kidney lysate for the expression of ATP5a and ATP5b. (E) Representative immunochemistry for ATP5a and ATP5b in kidneys. ****: $p<0.0001$.

Supplementary Figure 2. Detection of the efficiency of P2X7 receptor siRNA transfection in HK2 cells. (A) Representative immunoblots and (B) aggregate densitometric quantification of cell lysate for the expression of P2X7 receptor. **: $p<0.01$, ***: $p<0.001$.

Supplementary Figure 3. Renal IRI increased the expression of P2X7R and NLRP3 proteins in CD11b positive and E-cadherin positive cells. Representative immunofluorescent staining for (A) CD11b (red) and P2X7R (green), for (B) E-cadherin (red) and P2X7R (green), for (C) CD11b (red) and NLRP3 (green), and for (D) E-cadherin (red) and NLRP3 (green) merged with DAPI.

Supplementary Figure 4. Confirmation of the various chimeras by PCR for *P2X7r* gene in the tail and peripheral blood. We constructed radiation-induced BM chimeras between P2X7R (-/-) and P2X7R (+/+) mice. The genotyping was performed in the tail and peripheral blood of the bone marrow chimeras.

Supplementary Figure 5. NLRP3 inflammasome was activated in ischemic kidneys and in HK2 cells suffering from hypoxia/reoxygenation or ATP

stimulation. For A and B, mice were grouped into the Sham and the Ischemic-Reperfusion (n=5 per group). Representative immunofluorescent staining for (A) P2X7 receptor (green) and NLRP3 (red); for (B) NLRP3 (green) and ASC (red), merged with DAPI (blue). For C and D, Representative immunoblots of cell lysate for the expression of cleaved caspase-1, NLRP3 and ASC proteins in response to (A) hypoxia for 24 hours/reoxygenation for indicated time and to (B) ATP at different concentrations.
