# Supplementary figures and images for: P2X7 receptor signaling promotes inflammation in renal parenchymal cells suffering from ischemia-reperfusion injury
Source: Cell Death Dis. 2021 Jan 27;12(1):132. doi: 10.1038/s41419-020-03384-y (PMC7841183; doi:10.1038/s41419-020-03384-y)

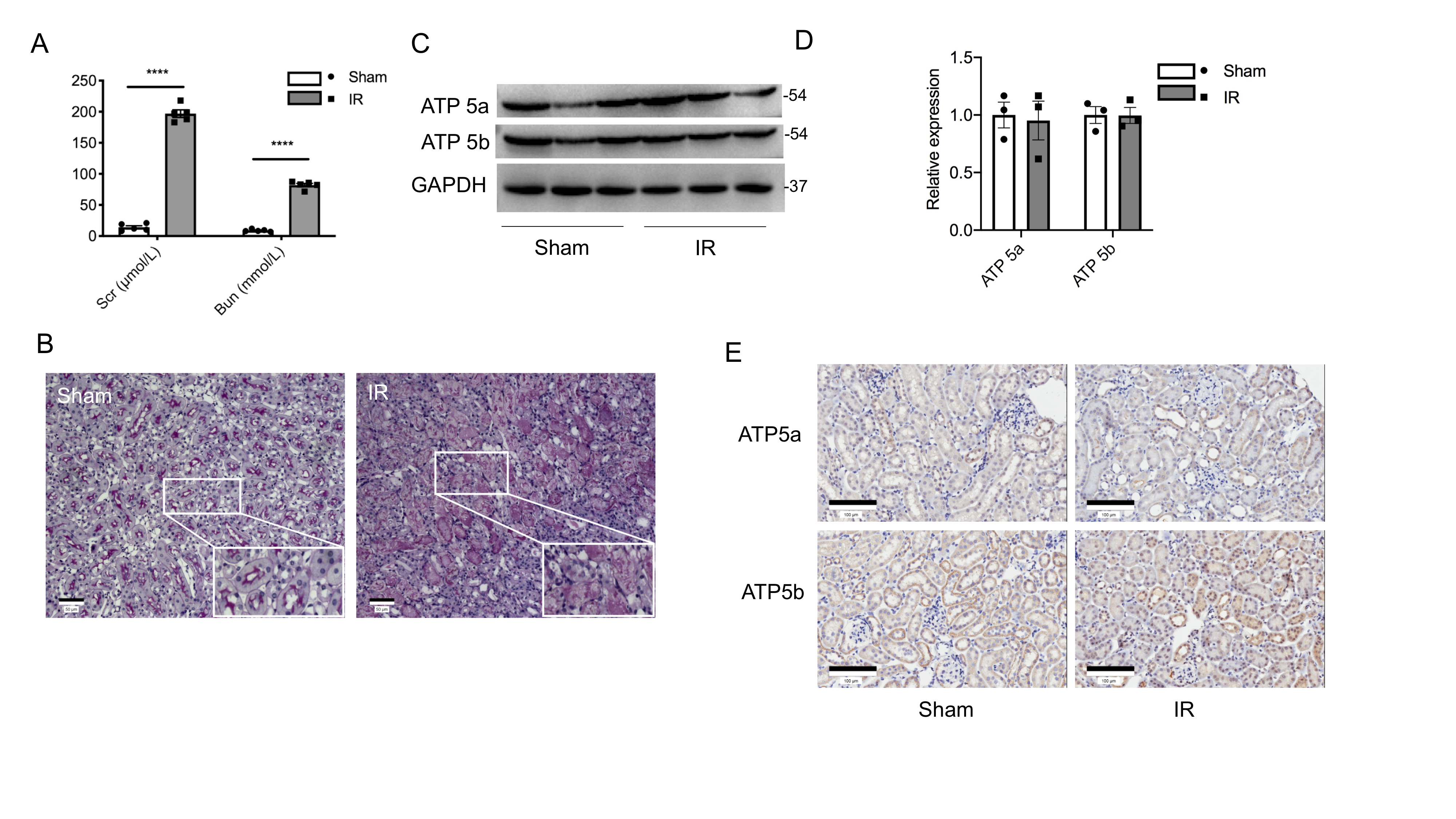

Supplement: Supplementary file 3 — Supplementary figure 1 [file 41419_2020_3384_MOESM3_ESM.tif]

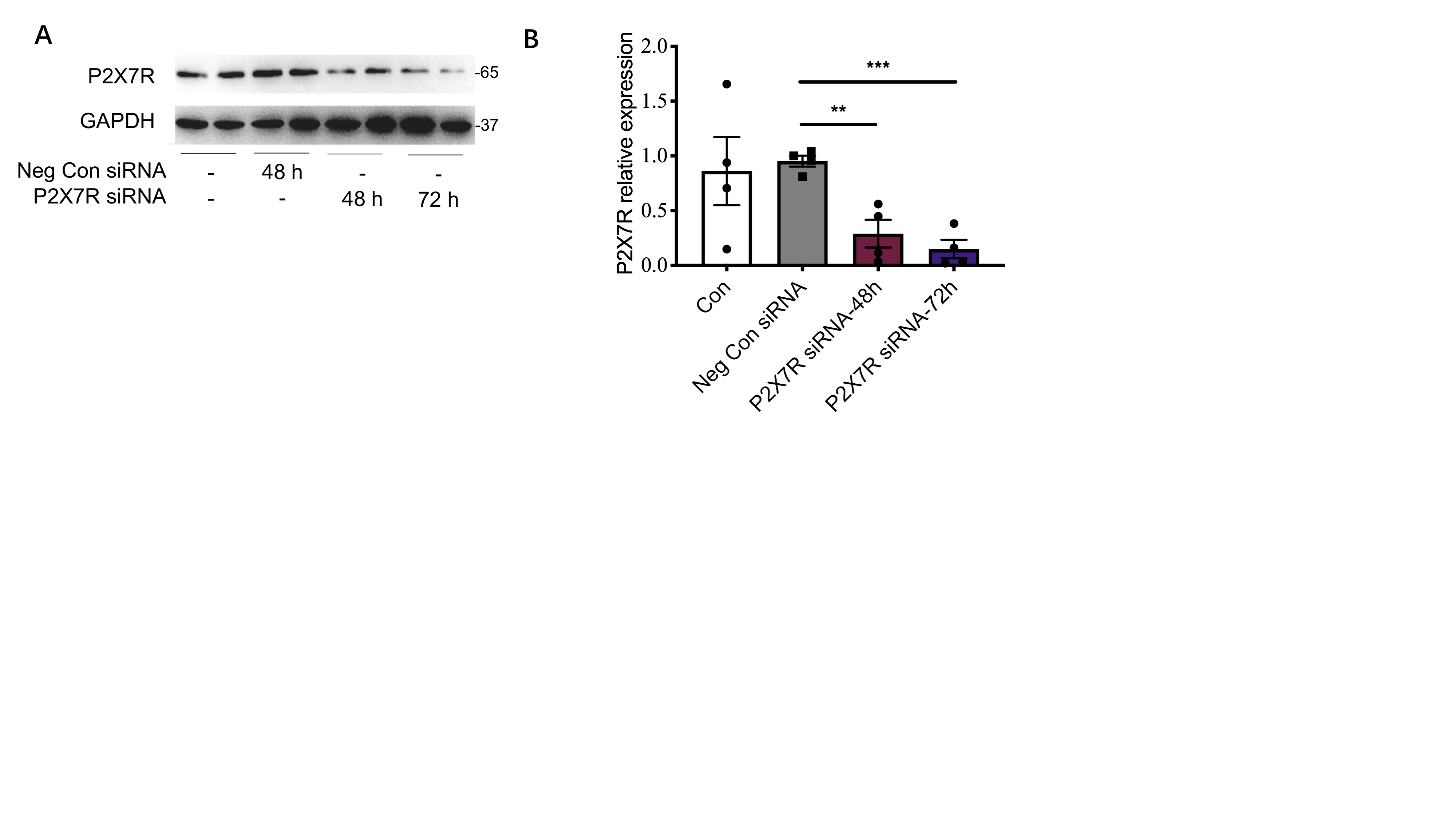

Supplement: Supplementary file 4 — Supplementary figure 2 [file 41419_2020_3384_MOESM4_ESM.tif]

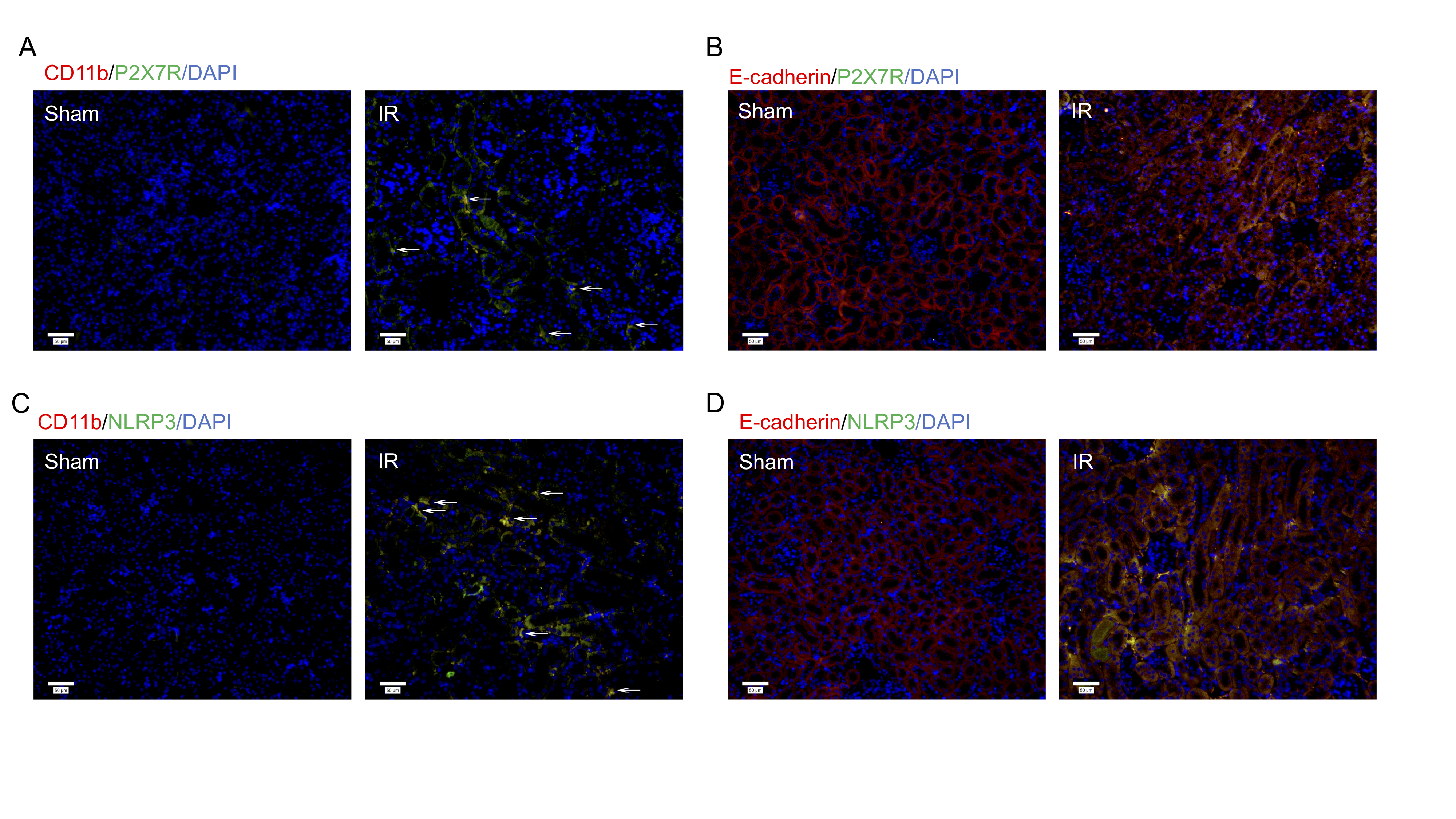

Supplement: Supplementary file 5 — Supplementary figure 3 [file 41419_2020_3384_MOESM5_ESM.tif]

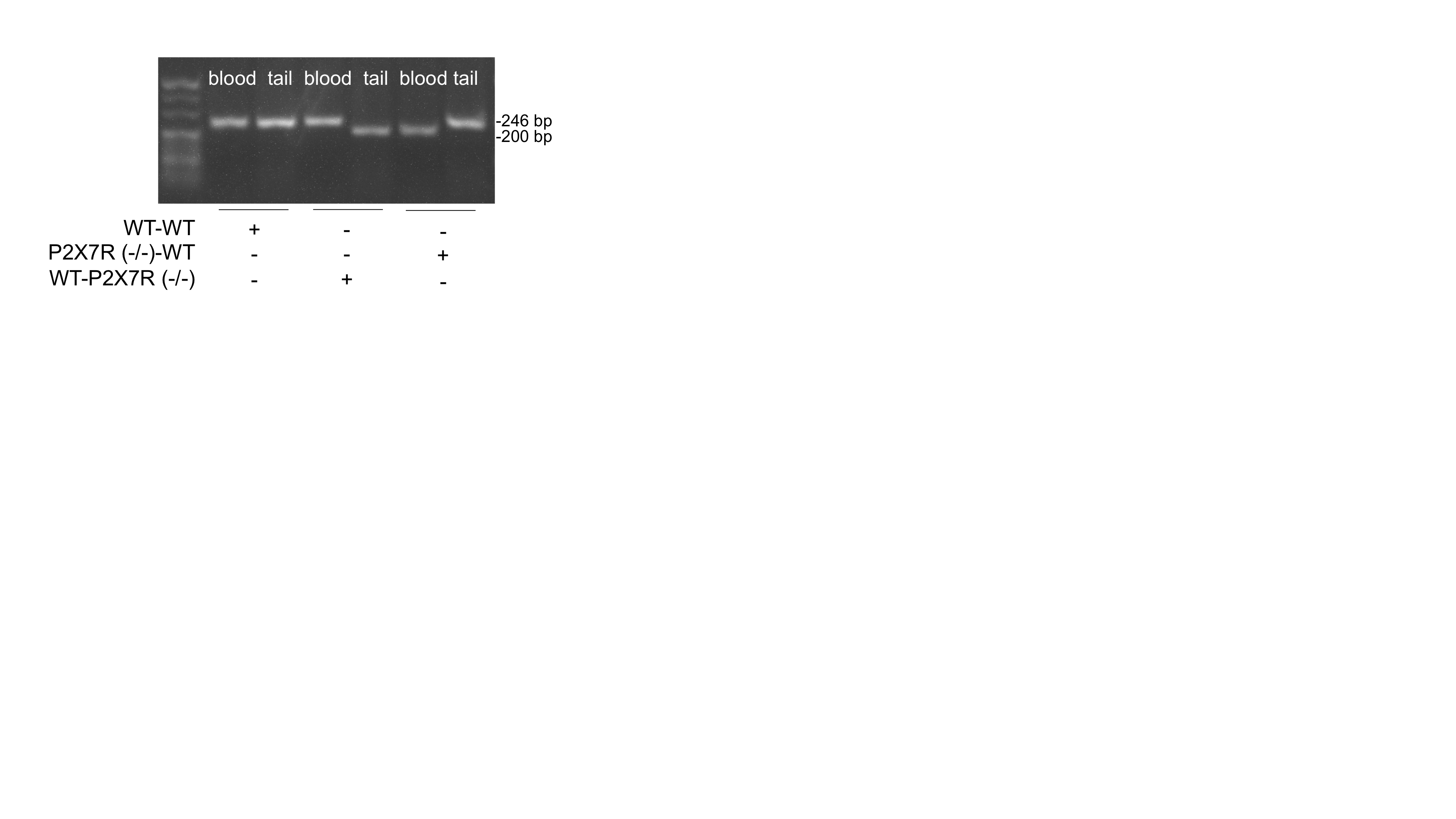

Supplement: Supplementary file 6 — Supplementary figure 4 [file 41419_2020_3384_MOESM6_ESM.tif]

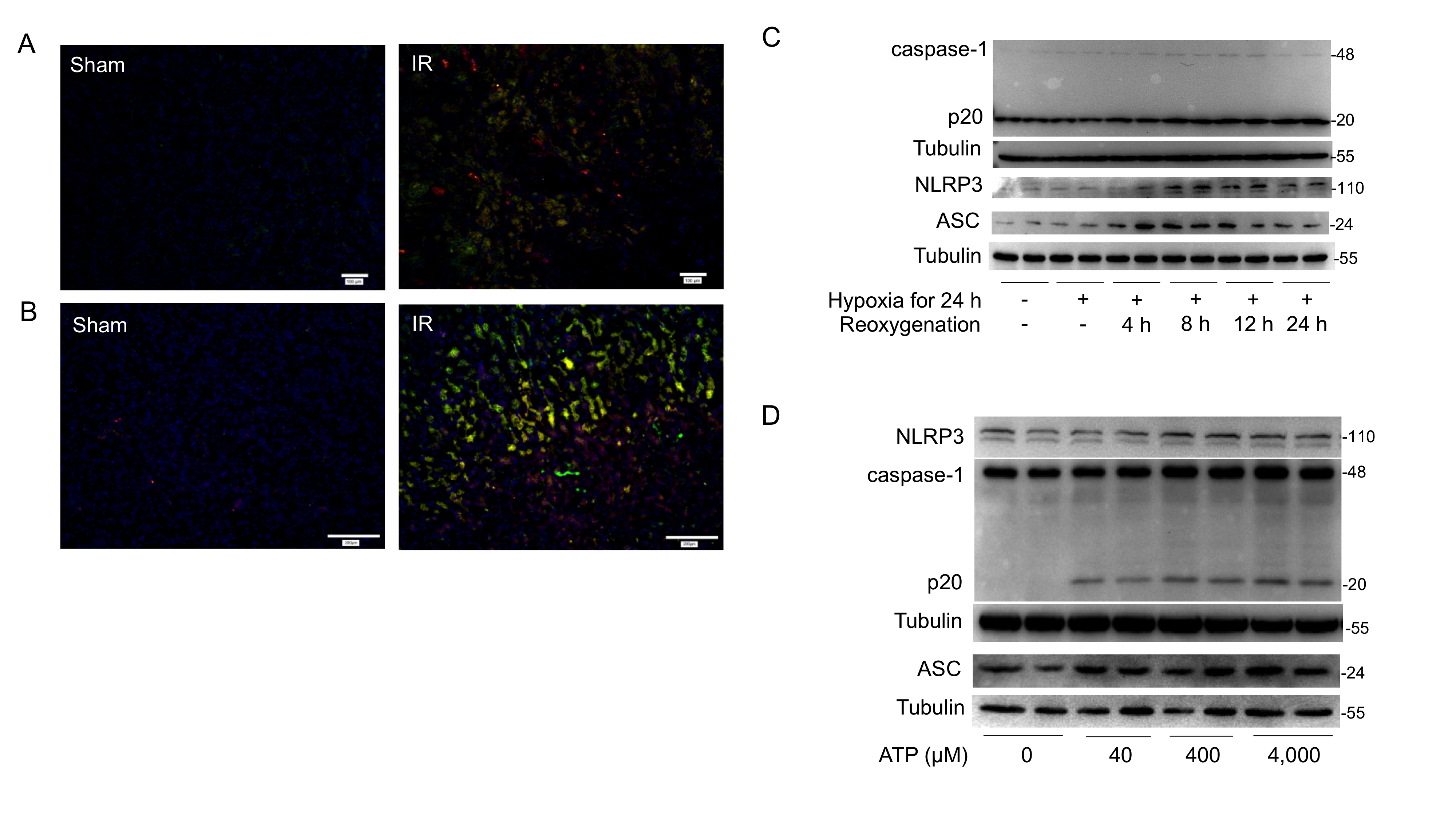

Supplement: Supplementary file 7 — Supplementary figure 5 [file 41419_2020_3384_MOESM7_ESM.tif]
